# Epigenetic Functions of SMYD5 and Its Role in Development, Cancer and Other Cellular Processes

**DOI:** 10.3390/ijms27135884

**Published:** 2026-06-30

**Authors:** Daniela Boehm, Kanika Khanna, Zichong Li, Melanie Ott

**Affiliations:** 1 Gladstone Infectious Disease Institute, Gladstone Institutes, San Francisco, CA 94158, USA; 2 Department of Medicine, University of California San Francisco, San Francisco, CA 94143, USA; 3 Chan Zuckerberg Biohub, San Francisco, CA 94158, USA

**Keywords:** SMYD5, RPL40, H3K36, H3K37, H4K20, development, cancer, HIV-1

## Abstract

The lysine methyltransferase SMYD5 is an important regulator of development and has been implicated in multiple malignancies, such as heart disease, lung and gastric cancers, breast and hepatocellular carcinomas, and inflammatory bowel disease. Further, SMYD5 has been linked to the mild hypothermia response, RNA translation, and HIV-1 transcription. SMYD5 is ubiquitously expressed in lymphocytes and the fetal brain, retina, heart, gut, liver, and reproductive organs. Mechanistically, SMYD5 methylates histone residues H3K36, H3K37, and H4K20, as well as non-histone targets such as the ribosomal protein RPL40 and the HIV-1 Tat protein. Here, we review the literature on SMYD5, focusing on its epigenetic functions and its roles in development, cancer, and other biological processes.

## 1. Introduction

SMYD5 is a member of the SET and MYND domain-containing (SMYD) family of protein lysine methyltransferases, which consists of five members in humans and mice (SMYD1-5). The split conserved catalytic SET domain—named after chromatin regulators in Drosophila [Su(var)3–9, Enhancer of zeste, and Trithorax]—is shared among most histone methyltransferases and mediates methylation of lysine residues [1,2]. The MYND domain—named after Myeloid translocation protein 8, Nervy, and DEAF-1—consists of a zinc finger motif that primarily mediates protein–protein interactions [3,4,5].

Over the last 15 years, SMYD5 has emerged as an important regulator of stem cell differentiation and is implicated in multiple diseases, including cardiovascular disease, various cancers (lung, gastric, breast, and hepatocellular), and inflammatory bowel disease. SMYD5 is also involved in physiological responses such as mild hypothermia and in other cellular processes like RNA translation and HIV-1 transcription. SMYD5 exerts its function by methylating several targets, including histone H3 at lysine 36 and 37 (H3K36me1, H3K37me1, H3K36me3), and by trimethylating histone H4 at lysine 20 (H4K20me3) [6,7,8]. In addition to histones, SMYD5 modifies the non-histone protein HIV-1 Tat [9]. Moreover, SMYD5 is currently the only known lysine methyltransferase capable of methylating the ubiquitin-ribosomal protein eL40 fusion protein RPL40 [10,11,12].

In this review, we will summarize the literature on SMYD5. Many publications related to SMYD5 have focused on its involvement in development and different types of cancers, but we also summarize its role in a broad range of cellular functions.

## 2. Discovery and Structure of SMYD5

SMYD5, also known as ZMYND23, retinoic acid-induced protein (RAI15), RRG1, or protein NN8-4AG, is located on chromosome 2 in the 2p13.2 region of the human genome and was discovered by M. Shago and V. Giguére in 1996 [13]. In their study, fragments derived from CpG islands of the murine genome were selected using gel mobility shift assays with in vitro-transcribed and -translated ligand-inducible retinoic acid and retinoid receptors [13]. The mouse NN8-4AG transcript, later named SMYD5, was upregulated by retinoic acid in F9 embryonal carcinoma cells and was homologous to an expressed sequence tag (EST41159) derived from a human infant brain cDNA library [13].

**Figure 1:**
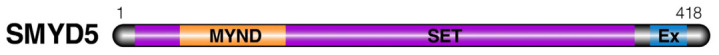
Domain structure of SMYD5.

Phylogenetic analysis of SMYD protein sequences in metazoans revealed that *Smyd* genes can be categorized into three main classes: *Smyd3* (which includes chordate-specific *Smyd1* and *Smyd2* genes), *Smyd4*, and *Smyd5* [14]. Among these, *Smyd5* is the most evolutionarily conserved, with one representative found across all metazoan species analyzed [14]. SMYD5 is, based on the structure of the catalytic domain, grouped into the class V-like SAM-binding methyltransferase superfamily, which includes the SET-domain, which is flanked by pre- and post-SET domains [15]. SMYD5 is the most unique SMYD family protein in vertebrates, as it lacks the tetratricopeptide repeat (TPR) motifs present in SMYD1–4, and instead only has a C-terminal glutamate-rich extension (Ex) (Figure 1). According to ensembl.org the *Smyd5* gene produces nine different splice variants, three of which are predicted to encode proteins (isoforms SMYD5-202, -204, and -209) [16]. The 2554 base pair (bp) SMYD5 isoform 1 (SMYD5-202, Q6GMV2) encodes a 418 amino acid (aa), a 47.3 kilo Dalton (kDa) protein that is considered the full-length wild type [16]. SMYD5 isoform 2 (SMYD5-204, C9IYN9) is missing aa 1–32 at the 5’UTR and encodes a 138 amino acid, 15.8 kDa, protein [16]. SMYD5 isoform 3 (SMYD5-209, F8WEJ9) encodes an 81 amino acid, 9 kDa, protein that is missing aa 82–418 of isoform 1 [16]. The biological function of the two short SMYD5 isoforms produced by alternative splicing has not been characterized thus far.

Several point mutations of SMYD5 that affect its enzymatic activity have been identified, including H316, C317, Y351 and Y372 [6,12,17].

A crystal structure of SMYD5 is still lacking. However, a recent study using AlphaFold shed some light on its unique structure and how it correlates with its functional divergence [18]. In this study, Zhang et al. validated AlphaFold’s reliability by comparing its model to known crystal structures of SMYD family proteins and subsequently identified a novel non-classical nuclear localization signal at the N-terminus of SMYD5 [18]. SMYD5 exhibits both cytoplasmic and nuclear localization, but recent studies suggest its primary function, particularly in cancer, is centered in the cytoplasm, where it acts as a methyltransferase targeting the ribosomal protein L40 (RPL40) at K22. In HeLa and Huh7 cells, endogenous SMYD5 is predominantly localized to the cytoplasm and in hepatocellular carcinoma cells; cytoplasmic SMYD5 is often upregulated and correlates with poor prognosis [11]. However, it possesses a novel, non-classical nuclear localization signal (NLS) at its N-terminus that may be able to drive its localization to the nucleus [18]. Several studies suggest that SMYD5 is present in the nucleus and acts as a histone methyltransferase (H3K36me3, H4K20me3) at promoters, mediating gene expression. In addition, multiple studies showed that SMYD5 binds to DNA, particularly to specific genomic regions to regulate gene expression [7,8,9,17]. SMYD5 binds to the promoters of Toll-like receptor 4 (TLR4), which target genes and repetitive DNA elements (such as LINE and LTR retrotransposons) [8,17]. By binding to these regions, SMYD5 helps establish a repressive chromatin state, specifically by mediating H4K20me3 to maintain heterochromatin stability. Further, SMYD5 is recruited to chromatin by RNA polymerase II (Pol II) to methylate H3K36 at promoters [7]. In addition, SMYD5 is recruited to promoter regions, such as the HIV-1 promoter, where it acts as a direct repressor [9]. Structural analysis (AlphaFold) indicates that the SMYD5 structure contains a unique, large, negatively charged “crab-like” structure, which may enable interactions with basic molecules such as protamines, which are histone substitutes involved in chromatin condensation during spermatogenesis [18]. In addition, within the SMYD family of methyltransferases (e.g., SMYD3), the MYND domain is associated with low direct DNA-binding ability [19]. However, SMYD5 is often recruited to chromatin in association with other complexes, such as NCoR (nuclear receptor corepressor), and some studies suggest it may not have direct, intrinsic DNA-binding ability [8]. Moreover, SMYD5s activity on histones is considered low or weak by some studies compared to its activity on RPL40, suggesting SMYD5 primarily performs its function in the cytoplasm [10,18].

To gain functional insights into the SMYD protein family, Abu-Farha et al. generated a protein interaction network for the three human SMYD proteins: SMYD2, SMYD3, and SMYD5 [20]. Using HEK293T cells expressing SMYD-Flag constructs, they performed Flag immunoprecipitation followed by mass spectrometry to identify interacting partners. In this study, SMYD5 interacts with nine proteins, four of which (NPM1, TOP1, GNL3, RUVBL2) also interact with SMYD2 and SMYD3. These four proteins are associated with DNA repair and chromatin maintenance during the cell cycle, notably through the regulation of p53 [20]. Since this first publication, the number of proteins interacting with the SMYD5 protein has expanded to 77 (genecards.org). In addition, three proteins, TRIM25, NXF1 and MOV10 have been identified as SMYD5 RNA interaction partners [21,22].

## 3. Biological Roles of SMYD5

### 3.1. Epigenetic Functions of SMYD5

**Figure 2:**
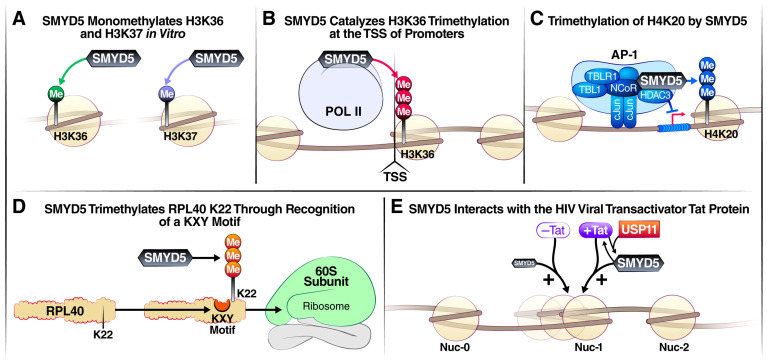
Selected mechanisms of action of SMYD5. (**A**) SMYD5 monomethylates histone H3 at lysines 36 and 37 (H3K36me1/H3K37me1) in vitro. (**B**) SMYD5 is recruited to chromatin by RNA polymerase II where it catalyzes histone H3 lysine 36 trimethylation (H3K36me3) at the transcription start site of promoters. (**C**) SMYD5 trimethylates histone 4 lysine 20 (H4K20me3), and in cooperation with nuclear receptor corepressor 1 complex (NCOR1), silences Toll-like receptor 4 (TLR4) pathway target genes in macrophages. (**D**) SMYD5-mediated RPL40 trimethylation at lysine 22 regulates mRNA translation. Loss of SMYD5, and consequently RPL40K22me3, reduces translational output and disrupts elongation. SMYD5 trimethylates RPL40 K22 through recognition of a KXY motif within RPL40 to catalyze this modification. (**E**) SMYD5 associates in vivo with the HIV promoter and interacts with the HIV trans-activation response (TAR) element RNA and the viral transactivator Tat protein. Upon binding, SMYD5 methylates Tat, an event that enhances Tat-mediated transcriptional activation of viral genes.

The epigenetic functions of SMYD5 in mammalian tissues have been described in only a limited number of studies. Here, we review the SMYD5 substrates published for SMYD5 and their biological outcome (Figure 2, Table 1).

### 3.2. Substrates in Human Cells

#### 3.2.1. Histone 3

SMYD5 monomethylates histone H3 at lysines 36 and 37 (H3K36me1 and H3K37me1) in vitro [6]. Using site-mutagenesis, Aljazi et al. identified histidine H316 of SMYD5 as a critical amino acid required for its methyltransferase activity. H316 is conserved in human, mouse, *Xenopus*, zebrafish and *Drosophila* and was previously reported as one of two amino acids whose mutation abolishes the enzymatic activity of SMYD5 [6,17]. These initial in vitro findings were subsequently validated in murine embryonic stem cells (mESCs). Crispr/Cas9-mediated genetic deletion of *Smyd5* partially reduced global H3K37me1 levels, but did not affect H6K36me1 levels, thereby suggesting that SMYD5 is one of several histone methyltransferases catalyzing histone H3K37me1 in vivo [6].

Another study in mESCs showed that SMYD5 catalyzes histone H3 lysine 36 trimethylation at promoters [7]. H3K36me3, typically deposited onto chromatin following RNA polymerase II (Pol II) elongation, marks active gene bodies with a gradient increasing from the 5′ end to the 3′ end [23]. Using CUT & Tag screening in *Setd2* knockout mESCs the authors found that, in addition to gene bodies, H3K36me3 is enriched at promoters in primary cells. They identified SMYD5, recruited to chromatin by Pol II, as the methyltransferase catalyzing H3K36me3 at promoters [7]. Functional studies showed that the deletion of SMYD5s C-terminal glutamic-acid-rich domain reduces the enzymatic activity of SMYD5 by loss of binding to histone core octamers and to H3–H4 tetramers. Overexpression of full-length *Smyd5*, but not the C-terminal domain-truncated *Smyd5*, restores H3K36me3 at promoters in *Smyd5*-knockout cells [7]. Together, these findings identify SMYD5 as a H3K36me3 methyltransferase at promoters, suggesting broader functions in chromatin regulation and gene expression [7].

Based on recent research, SMYD5 does not primarily target H3K9, but its function is often linked to the H3K9 methylation pathway. SMYD5 works in conjunction with heterochromatin proteins like HP1α and H3K9 methyltransferases, specifically by interacting with G9a, to promote the silencing of endogenous retroviruses (ERVs) and maintaining heterochromatin [8].

#### 3.2.2. Histone 4

Two studies reported histone 4 lysine 20 (H4K20) as a potential SMYD5 substrate [8,17]. Stender et al. showed that SMYD5 associates with the nuclear receptor corepressor (NCoR) complex [8]. This mechanism acts as a negative inflammatory response to prevent Toll-like receptor 4 (TLR4) activation by depositing H4K20me3 marks at promoters in macrophages [17]. Kidder et al. showed that this methylation occurs at repetitive LINE/LTR DNA sequences that recruit SMYD5 [8,17].

#### 3.2.3. RPL40

Ubiquitin-ribosomal protein eL40 fusion protein, RPL40, is a component of the 60S ribosomal subunit, encoded by the human precursor *UBA52* gene. Cleavage of the UBA52 precursor protein by deubiquitinases (DUBs) releases the N-terminal monoubiquitin, producing the 52 amino acid RPL40 protein, which incorporates into the pre-60S subunit after its export to the cytoplasm [24]. In 1997, Williamson et al. reported that rat RPL40/eL40 is trimethylated at lysine 22 [25], and 26 years later, Holm et al. confirmed this modification in human ribosomes [26]. However, the enzyme responsible for RPL40 K22 methylation was unknown until recently, when three different research groups demonstrated SMYD5 to be the responsible methyltransferase [10,11,12]. While lysine methylation is well known for regulating transcription, its role in translation remains less understood. Park et al. first showed that SMYD5-mediated RPL40 trimethylation regulates mRNA translation in malignant gastric adenocarcinoma (GAC) progression [10]. Using a CRISPR-guided biochemical–proteomics approach, they demonstrated that SMYD5 generates endogenous RPL40K22me3, which in turn regulates translation elongation rates and overall protein synthesis [10].

Miao et al. corroborated these findings, showing that SMYD5 has robust in vitro activity toward RPL40 K22, primarily catalyzing RPL40 K22me3 in cells [11]. Loss of SMYD5, and consequently RPL40K22me3, reduces translational output and disrupts elongation, as evidenced by increased ribosome collisions [11].

A third study reported that SMYD5 trimethylates RPL40 K22 through recognition of a KXY motif within RPL40 to catalyze this modification [12]. Mass spectrometry identified RPL40 K22 as one of 12 primary ribosomal methylation sites in K562 cells. Recombinant SMYD5 has robust in vitro activity against this site, and active site mutations ablate methyltransferase function [12]. Knockout of SMYD5 in K562 cells results in complete loss of RPL40 K22 methylation and decreased polysome levels [12]. Additionally, this study provided an AlphaFold2 model of SMYD5 with bound S-adenosylmethionine, showing that the deletion of essential components of SMYD family proteins such as the “M-insertion” [residues 80–101] and “S-insertion” [residues 260–298]) abolished its catalytic activity [12].

#### 3.2.4. Viral Substrates

SMYD5 is an activator of human immunodeficiency virus 1 (HIV-1) transcription [9]. It is highly expressed in CD4^+^ T cells. The depletion of SMYD5 in HIV latently infected T-cell lines and primary CD4^+^ T cells represses latent HIV-1 reactivation [9]. Mechanistically, SMYD5 associates in vivo with the HIV promoter and interacts with the HIV trans-activation response (TAR) element RNA and the viral transactivator Tat protein. Upon binding, SMYD5 methylates Tat, an event that enhances Tat-mediated transcriptional activation of viral genes [9,27]. In turn, SMYD5 protein levels increase in cells expressing Tat. This process requires expression of the Tat cofactor, ubiquitin-specific peptidase 11 (USP11), which stabilizes SMYD5 by preventing its degradation. Collectively, SMYD5 is a host factor that promotes HIV-1 transcription and is itself stabilized by Tat and USP11 [9].

## 4. Role of SMYD5 in Development

The developmental functions of SMYD5 have been explored across multiple vertebrate models, particularly zebrafish. During early embryogenesis, SMYD5 expression is high and declines steadily over time [28]. Loss-of-function studies of SMYD5 in zebrafish embryos demonstrated disruption of normal blood cell development and increased expression of markers of hematopoiesis, including *pu.1*, *mpx*, *cymb*, and *l-plastin* [28].

SMYD5-mediated regulation of heterochromatin via H4K20me3 has been linked to pluripotency and chromosome integrity in embryonic stem cells [29]. Eliminating SMYD5 disrupted self-renewal by altering *Oct4* gene levels affecting differentiation [29].

SMYD5 also plays a role in skeletal myofibrillogenesis in a zebrafish model of copper ion (Cu^2+^) imbalance. Unbalanced copper homeostasis is associated with developmental defects in vertebrate myogenesis [30]. Cu^2+^-stressed zebrafish embryos and larvae showed reduced locomotor speed, as well as loose and decreased myofibrils in skeletal muscles. In copper stressed embryos, whole-genome DNA methylation analysis unveiled that the *Smyd5* gene exhibited significant promoter hyper-methylation, which lead to increased and abnormal *Smyd5* expression. These data suggest a link between unbalanced copper homeostasis with specific gene promoter methylation and epigenetic histone modifications, which may affect signal transduction and lead to the myofibrillogenesis defects [30].

Further evidence of involvement of *Smyd5* in development and reproduction came from a study in the self-fertilizing mangrove rivulus fish, *Kryptolebias marmoratus* [31]. Investigation of evolutionary relationships with other animal methyltransferases and characterization of expression patterns during embryonic development and in adult tissues identified *Smyd5* as one of 48 lysine methyltransferase orthologues [31]. Domain conservation and expression profiles suggest that they might play important roles during development, gametogenesis and neurogenesis [31].

SMYD5 has also been implicated in stem cell maintenance. Kidder et al. demonstrated that loss of SMYD5 in mice alters embryonic stem cell differentiation [17]. Indeed, a *Smyd5* knockout murine embryonic stem cell line exhibited abnormal embryonic stem cell colony morphology and a remarkable reduction in alkaline phosphatase staining, an undifferentiated embryonic stem cell marker. Embryoid body assays using the same SMYD5 knockout line cells led to dysregulated gene expression and decreased global H4K20me3 levels, whereas overexpression of *Smyd5* reversed these phenotypes. Based on these and related experiments, the authors proposed that SMYD5 regulates embryonic stem cell maintenance by silencing lineage-specific genes [17]. In extended duration embryoid body assays, *Smyd5* knockout cells underwent malignant transformation, and injection of these cells into immunosuppressed mice led to tumor formation, supporting the role of SMYD5 as a tumor suppressor [29].

## 5. Impact of SMYD5 in Cancers

SMYD5 levels are elevated in several types of cancers. In the following sections, we discuss the role of SMYD5 in different cancer types.

### 5.1. Breast Cancer

Increased SMYD5 mRNA levels have been reported in specific breast cancer sub-types, as well as lower SMYD5 expression is associated with improved relapse-free survival in breast cancer patients [32]. A comparison of the transcriptional data for SMYD1-5 with the survival data of breast carcinoma patients from multiple datasets—ONCOMINE, Breast Cancer Gene-Expression Miner v4.0, Kaplan–Meier Plotter, The Cancer Genome Atlas and cBioPortal—demonstrated an increase in the SMYD2/3/5 mRNA levels in breast carcinoma tissues compared with normal tissue [32]. Further, SMYD5 mRNA levels are decreased in patients with estrogen receptor (ER)/progesterone receptor (PR)-positive breast cancers compared with ER/PR-negative cases, but increased in patients with HER2-positive breast cancer [32]. SMYD5 mRNA levels were also increased in patients with triple-negative breast cancer, an aggressive form of breast cancer lacking ER, PR, and HER2 expression [32]. In addition, increased SMYD5 mRNA levels were associated with advanced Scarff–Bloom–Richardson grade, an important prognostic indicator in breast cancer [32]. These findings suggest a potential role for SMYD5 in the progression of breast cancer [32].

### 5.2. Gastric Cancer

SMYD5 is associated with gastric cancer, with higher SMYD5 expression correlating with poorer overall survival in patients [33]. Bioinformatics analysis of the expression of histone modification-associated genes in gastric cancer and normal tissues from Oncomine, the Gene Expression Omnibus (GEO) and The Cancer Genome Atlas (TCGA) datasets demonstrated that SMYD5 was upregulated in gastric cancer, with aberrant expression evident in gastric intestinal-type adenocarcinoma [33].

A recent study further established the link between SMYD5 and gastric cancer [10]. Trimethylation of RPL40 at lysine 22 by SMYD5 regulates mRNA translation to promote malignant progression of gastric adenocarcinoma with lethal peritoneal ascites [10]. In vivo, SMYD5 knockout in familial and sporadic mouse models of malignant gastric adenocarcinoma blocks metastatic disease, including peritoneal carcinomatosis. Suppressing SMYD5-mediated methylation of RPL40 inhibits growth of human cancer cells and patient-derived gastric adenocarcinoma xenografts, rendering them hypersensitive to PI3K and mTOR inhibitors (e.g., omipalisib) [10]. Notably, combining SMYD5 depletion with PI3K-mTOR inhibition and chimeric antigen receptor (CAR)-T-cell therapy cures an otherwise lethal in vivo mouse model of aggressive gastric adenocarcinoma-derived peritoneal carcinomatosis [10].

### 5.3. Hepatocellular Carcinoma

SMYD5 has been identified as a biomarker for liver hepatocellular carcinomas, with high expression associated with poor patient prognosis [7,34]. Gene set enrichment analysis (GSEA) of the TCGA datasets suggested that complement and coagulation cascades, fatty acid metabolism, primary bile acid biosynthesis, drug metabolism, cytochrome P450, PPAR signaling pathway, and retinol metabolism were differentially enriched in the SMYD5 high-expression phenotype [34]. Further, SMYD5 upregulation in hepatocellular carcinoma cells is induced by promoter hypo-methylation, and SMYD5 silencing abrogates cell proliferation, migration, and invasion, while enhancing paclitaxel sensitivity in hepatocellular carcinoma [34].

Further, Miao et al. reported that SMYD5 and RPL40 K22me3 are upregulated in hepatocellular carcinoma and negatively correlated with patient prognosis [11]. This study in genetically engineered mouse and patient-derived xenograft (PDX) models corroborates the finding by Park et al. that depleting SMYD5 renders cancer cells hypersensitive to mTOR inhibition [11].

Elevated expression of SMYD5 in hepatocellular carcinoma tissues compared to normal liver tissues was also observed by Hu et al. [35]. Their study confirmed that high levels of SMYD5 correlate with poor overall survival and disease-free survival rates in hepatocellular carcinoma patients [35]. Here a link between loss of SMYD5, inhibition of cell proliferation and increased apoptosis in hepatocellular carcinoma cell lines is shown [35]. Additionally, the authors suggest an interaction between SMYD5 and BRD4. However, the authors appear to draw conclusions from western blotting of BRD4 based on a nonspecific band. Conrad et al. showed that the short isoform of BRD4 is a protein of approximately 74 kDa, the molecular weight of the long isoform is 200 kDa [36]. A nonspecific band at approximately 150 kDa is resistant to BRD4 shRNA and appears on all BRD4 western blots [36].

### 5.4. Lung Cancer

Metastasis is the main cause of death in lung cancer patients, and hence, major therapeutic efforts are directed toward its suppression. Recently, SMYD5 has been identified as a novel regulator of metastasis in lung cancer [37]. Tae et al. found SMYD5 overexpression in lung cancer based on both RNA-sequencing analysis from the TCGA portal and immunohistochemical studies [37]. SMYD5 knockdown inhibits cell migration and invasion in NCI-H1299 and H1703 cell lines by changing expression levels of epithelial–mesenchymal transition (EMT) markers and MMP9 [37]. SMYD5 knockdown also increased Src homology 2-B3 expression by decreasing H4K20 trimethylation [37]. Furthermore, in an in vitro EMT model induced by TGF-β, SMYD5 knockdown reduced cell migration and invasion in the highly invasive NCI-H1299 and H1703 cell lines [37].

SMYD5 downregulation is a notable feature of alveolar macrophage responses to multiwalled carbon nanotube (MWCNT) exposure, implicating SMYD5 in macro-phage-mediated immune responses to nanomaterial inhalation [38]. Erdem et al. investigated the immunomodulating properties of two industrially relevant nanomaterials, nanocellulose and MWCNTs, using MH-S alveolar macrophages at the air–liquid interface. Cells were exposed to cellulose nanocrystals (CNC), cellulose nanofibers (CNF) and two MWCNT types (NM-400 and NM-401). Following exposure, changes in macrophage polarization markers, inflammatory cytokines, and epigenetic regulation were accessed [38]. While CNF exposure leads to enhanced M1 phenotype, MWCNTs promote M2 phenotype. Furthermore, MWCNTs exposure induced prominent epigenetic alterations, including changes in the expression of histone modification, DNA methylation enzymes, as well as in miRNA transcript levels. MWCNT-enhanced changes in the macrophage phenotype also correlate with prominent downregulation of SMYD5 indicating that SMYD5 may have a role in the Th2 responses to MWCNT [38].

## 6. Other Functions of SMYD5

### 6.1. Thermal Stability of Proteins

Studying the thermal stability of proteins can reveal structural features and identify novel interaction partners, information that remains incomplete for SMYD5. To address this, Zhang et al. developed a machine learning strategy that combines orthogonal partial least squares regression and stability screening of the Silver Bullets Bio library to identify biologically active molecules that enhance SMYD5 stability [39]. Protamine, a histone substitute involved in chromatin condensation during spermatogenesis, emerged as the most potent stabilizer [39]. The structural stability of SMYD5 is regulated by its unique C-terminal poly-glutamic acid (poly-E) tract and a 30-residue insertion in the MYND domain. However, in the presence of protamine, these structural elements are unable to affect thermal stability. The stability-enhancing effect of protamine is SMYD5-specific. In contrast, protamine destabilizes SMYD2, a closely related homolog. Both SMYD5 and SMYD2 interact with protamine, but SMYD5 interaction is independent of the poly-E tract and M-insertion elements [39]. The observation that protamine enhances SMYD5 stability is intriguing given that protamines and histones are largely mutually exclusive during spermatogenesis: protamines replace histones on chromatin as sperm mature. One possible explanation is that this interaction reflects a transitional regulatory mechanism: during the early stages of spermiogenesis, before histone-to-protamine exchange is complete, SMYD5 may be active in chromatin regions still associated with histones, while protamine binding could serve to “prime” or stabilize SMYD5 for subsequent activity [39,40]. Alternatively, protamine binding to SMYD5 may modulate its activity in the small fraction of the genome (~4% in humans) that retains histones in mature sperm, which is enriched at developmentally important gene loci [41]. A third possibility is that protamine acts as a structural chaperone for SMYD5 independently of histone substrate availability, given the large negatively charged cleft in SMYD5’s unique structure that may serve as a binding site for basic proteins like protamines [18]. Future studies will be needed to determine whether SMYD5’s methyltransferase activity is directly modulated by protamine binding.

### 6.2. Mild Hypothermia Response

A CRISPR-Cas9 mutagenesis screen identified SMYD5 as an epigenetic gatekeeper of the mild hypothermia response [42]. Organisms have homeostatic mechanisms to respond to cold temperature to ensure survival, including the mammalian neuroprotective mild hypothermia response, which is activated at 32 °C. Mild hypothermia reduces SMYD5 levels both in vitro and in vivo via proteasomal degradation [42]. Notably, the mild hypothermia response could also be activated from euthermia (37 °C) by the FDA-approved drug entacapone [42]. During euthermia, SMYD5 represses the key mild hypothermia response gene *SP1* by depositing the repressive histone mark H3K36me3. This repression is relieved at 32 °C, enabling *SP1* activation. In addition to *SP1*, Rafnsdottir et al. identified 45 additional SMYD5-temperature dependent genes, suggesting a broader role of SMYD5 in regulating mild hypothermia response [42].

### 6.3. Inflammatory Bowel Disease (IBD)

Epithelial SMYD5 has been implicated in exacerbating inflammatory bowel disease (IBD) [43]. IBD, including Crohn’s disease and ulcerative colitis, are chronic disorders of the gastrointestinal tract [44]. Examination of SMYD5 expression in human colonic tissues via immunohistochemical staining demonstrates that SMYD5 is expressed mainly in colonic epithelia and lamina propria/stroma, with significantly higher levels in IBD patients with active inflammation compared to healthy controls [43]. Given the association of IBD with mitochondrial dysfunction, PGC-1α, a key regulator of mitochondrial activity, was also examined. Intestinal epithelia from both IBD patients and colitic mice exhibited elevated levels of SMYD5 and decreased levels of PGC-1α. SMYD5 depletion in colonic epithelia cells protected mice from dextran sulfate sodium-induced colitis. Mechanistically, SMYD5 attenuates mitochondrial functions in colonic epithelial cells and promotes IBD progression by lysine methylation of PGC-1α and its subsequent ubiquitination and degradation [43].

### 6.4. Rheumatoid Arthritis

A recent study suggests that SMYD5 is a dual regulator of synovial fibroblast homeostasis and rheumatoid arthritis (RA) pathogenesis [45]. RA is a chronic autoimmune disease that leads to progressive bone and cartilage damage [46]. Recently, fibroblast-like synoviocytes (FLSs) have emerged as a potential therapeutic target [47]. FLSs are specialized mesenchymal cells that line joint cavities and maintain healthy joints by producing lubricants such as hyaluronic acid, fibronectin and collagen [48].

In RA, activation of immune cells leads to excessive secretion of cytokines, including interleukin-1 (IL-1), tumor necrosis factor (TNF), and platelet-derived growth factor (PDGF). Further, SMYD5 expression is significantly elevated in the synovial tissues of RA patients and in IL-1β-induced FLS [45]. SMYD5 promotes FLS proliferation and inflammation by mediating posttranslational modifications and activating downstream signaling pathways. Xiao et al. showed that SMYD5 methylates forkhead box protein O1 (FOXO1), accelerating its degradation through ubiquitination. Additionally, SMYD5 promotes lactate production to activate NF-κB signaling pathways by upregulating hexokinases-2 (HK2) expression, a key glycolytic enzyme. The authors showed in a collagen-induced arthritis mouse model that knockdown of SMYD5 effectively alleviates joint swelling, bone erosion, and overall arthritis severity [45].

### 6.5. Heart Disease

SMYD5 is implicated in the regulation of histone H4K20 trimethylation in heart disease [49]. Hickenlooper et al. used immunoblotting and mass spectrometry and found that lysine trimethylation at histone H4 is differentially regulated in cardiomyocytes: it increases during acute hypertrophic stress in cell models and decreases during sustained ischemic injury and cardiac dysfunction in animal models [49]. Examination of publicly available data further identified SMYD5 as differentially expressed in heart failure patients, suggesting a potential role in modulating H4K20 methylation and contributing to cardiac pathology [49,50].

### 6.6. Maternal Prenatal Stressful Life Events and Childhood Traumatic Events

Recently, decreased expression of SMYD5 has been associated with maternal prenatal stressful life events (SLEs) and childhood traumatic events (CTEs) [51]. Prenatal exposure to maternal psychological stress is associated with increased risk for adverse birth and child health outcomes. Accumulating evidence suggests that pre-conceptional stress may also be transmitted intergenerationally with adverse impacts. To understand the mechanisms linking these exposures to offspring outcomes, particularly those related to the placenta, Baker et al. performed RNA sequencing in 1029 mother–child pairs from two birth cohorts in Washington state and Memphis, Tennessee [51]. Analysis of individual gene–SLE/CTE associations, combined with ensemble gene set enrichment across 11 methods revealed that higher numbers of prenatal SLEs were significantly associated with decreased expression of SMYD5 [51].

## 7. Conclusions

In conclusion, SMYD5 is a unique protein lysine methyltransferase with broad molecular functions. In contrast to other SMYD family members, SMYD5 is ubiquitously expressed in human tissue and cells, which might help explain the protein’s diverse functions [52]. During early embryogenesis, SMYD5 controls heterochromatin formation, maintains chromosome integrity during stem cell differentiation, and regulates primitive and definitive hematopoiesis [28,29]. SMYD5 is largely dispensable for morphological heart development, in stark contrast to family member SMYD1, which is critical for heart development and metabolism and maintenance of the adult heart [53,54]. However, studies on murine models have shown that loss of SMYD5 leads to elevated heart-to-body weight ratios and thickening of the ventricular wall, and it has therefore been proposed to serve as a crucial gatekeeper against pathological cardiac stress by inhibiting abnormal hypertrophic cell growth and cardiac fibrosis in the adult myocardium [55].

The limited number of studies available so far indicate that SMYD5’s primary role involves regulating gene expression and translation. SMYD5’s exact function depends on whether it operates in the cytoplasm or the nucleus. In the cytoplasm, SMYD5 acts as a ribosomal methyltransferase enhancing translation [10,11,12]. It specifically recognizes a KXY motif to catalyze the trimethylation of lysine 22 on RPL40 [12]. This methylation event is crucial for efficient translation elongation and overall protein synthesis capacity [10,11,12]. In the nucleus, SMYD5 plays multiple structural and epigenetic roles. It interacts with corepressor complexes to establish repressive chromatin states [29]. It generates methylation marks (such as H4K20me3) to maintain heterochromatin and silence transposable elements, safeguarding genome stability. In addition, SMYD5 acts as an epigenetic regulator in the nucleosome corepressor (NCoR) complex, repressing specific inflammatory genes (e.g., TLR4-driven responses) [8].

SMYD5 has been implicated in multiple biological processes and malignancies, such as heart disease, lung and gastric cancers, breast and hepatocellular carcinomas, inflammatory bowel disease and rheumatoid arthritis. While SMYD5’s exact clinical connection to inflammatory bowel disease and rheumatoid arthritis is still emerging, current studies established it as a critical epigenetic regulator in both immune-related and inflammatory diseases. In IBD, SMYD5 impairs healthy mitochondrial function by degrading PGC-1α, which exaggerates inflammation and compromises intestinal barrier integrity [43]. In RA, SMYD5 promotes post-translational modifications that activate downstream signaling pathways, leading to FLS proliferation and heightened joint inflammation [45]. In both diseases, knockdown of SMYD5 showed therapeutic potential, which led to protection of mice from experimental colitis in IBD and alleviated joint injury and improved synovial repair in RA [43,45].

In addition, SMYD5 is proposed to have a role in environmental adaptation by working as an “epigenetic gatekeeper” that responds to temperature cues, repressing certain genes during standard euthermia that are otherwise active during mild hypothermia [42]. At normal body temperatures (37 °C), SMYD5 is stable and active [42]. It deposits the H3K36me3 epigenetic mark at specific gene promoters, directly repressing key mild hypothermia response genes like SP1 [42]. The degradation of SMYD5 during cooling (32 °C) removes the transcriptional repression mark H3K36me3, driving the expression of over 30 temperature-sensitive, neuroprotective genes [18,42]. The degradation of SMYD5 at 32 °C is caused by the thermal unfolding of its M-insertion and S-insertion, which exposes hydrophobic residues [18]. At normal temperature, SMYD5’s structural integrity is characterized by stacking interactions between its M-insertion and S-insertion [18].

In cancer, most research indicates that SMYD5 generally acts as an oncogene (tumor promoter) rather than as a tumor suppressor in cancers like gastric adenocarcinoma (GAC), hepatocellular carcinoma (HCC), and lung cancer. High SMYD5 expression accelerates cancer cell growth, migration, and metastasis [37]. Limited scientific evidence points to ribosomal methylation as the primary oncogenic mechanism by which SMYD5 trimethylates the ribosomal protein rpL40. This modification enhances oncogenic gene translation, allowing aggressive tumor cells to thrive [10]. Elevated SMYD5 levels are heavily linked to poor clinical outcomes in patients with liver and stomach cancers [11]. Beyond that, SMYD5 also promotes tumorigenesis by enriching the active chromatin mark H3K36me3 at promoters [7]. Depleting SMYD5 has been shown to halt tumor growth, reduce metastasis, and sensitize malignant cells to mTOR inhibition [10]. However, limited scientific evidence shows that SMYD5 can also act as a tumor suppressor in mESCs as *Smyd5* knockout cells underwent malignant transformation [29]. Further studies are needed to determine whether SMYD5 truly displays both oncogenic and tumor suppressive properties or if these opposing findings depend on cellular context or experimental model system. Based on these findings, SMYD5 could serve as a potential therapeutic target for cancer treatment, and combining chemotherapy with SMYD5 inhibitor(s) or activators may enhance the therapeutic effect of cancer treatment.

Research of SMYD5 has uncovered diverse roles and biological functions in cancer, development and other diseases and has recently shifted the understanding of SMYD5s function from a traditional histone methyltransferase to a critical regulator of ribosomal translation. The future SMYD5 research agenda should aim to (1) resolve the crystal structure of SMYD5, (2) mechanistically determine the duality of SMYD5 activity on histones or ribosomes by clarifying the exact conditions under which it acts on chromatin remodeling versus active translation, (3) exploit the newly discovered SMYD5-rpL40 connection for cancer therapies and expand on the discovery that SMYD5 depletion sensitizes aggressive tumors to PI3K/mTOR pathway inhibitors, (4) investigate exactly how SMYD5 overexpression drives the epithelial–mesenchymal transition and metastatic behavior in cancers, and (5) develop a highly specific SMYD5 enzymatic inhibitor for therapy.

## Figures and Tables

**Table 1:** Histone and non-histone substrates of SMYD5.

Substrate	Modified Lysine Residue	Model	Method	References
Histone 3	H3K36me1	In vitro	Protein Auto-rad, MS	[6]
		In mESCs	WB	[6]
	H3K37me1	In vitro	Protein Auto-rad, MS	[6]
		In mESCs	WB	[6]
	H3K36me3	In mESCs	WB, Protein MS	[7]
Histone 4	H4K20me3	In vitro	Protein Auto-rad, WB	[8]
		In cells	ChIP	[8]
		In vivo in ESCs	WB, ChIP	[17]
RPL40	K22me3	In vitro	Protein Auto-rad, HPLC	[10]
		In cells	WB	[10]
		In vitro	Protein Auto-rad, MS, WB	[11]
		In cells and in mice	IP, WB, IHC staining	[11]
		In vitro and in cells	MS, PRM	[12]
HIV1 Tat	unknown	In vitro	Protein Auto-rad	[9]

Auto-rad: autoradiography; ChIP: chromatin immunoprecipitation; ESCs: embryonic stem cells; mESCs: mouse embryonic stem cells; HPLC: high-performance liquid chromatography; IHC: immunohistochemistry; IP: immunoprecipitation; MS: mass spectrometry; PRM: parallel reaction monitoring; and WB: western blot.

## Data Availability

No new data were created or analyzed in this study.

## References

[1] Dillon S.C., Zhang X., Trievel R.C., Cheng X. (2005). The SET-domain protein superfamily: Protein lysine methyltransferases. Genome Biol..

[2] Jenuwein T., Laible G., Dorn R., Reuter G. (1998). SET domain proteins modulate chromatin domains in eu- and heterochromatin. Cell. Mol. Life Sci..

[3] Ansieau S., Leutz A. (2002). The conserved Mynd domain of BS69 binds cellular and oncoviral proteins through a common PXLXP motif. J. Biol. Chem..

[4] Spellmon N., Holcomb J., Trescott L., Sirinupong N., Yang Z. (2015). Structure and function of SET and MYND domain-containing proteins. Int. J. Mol. Sci..

[5] Vilas C.K., Emery L.E., Denchi E.L., Miller K.M. (2018). Caught with One’s Zinc Fingers in the Genome Integrity Cookie Jar. Trends Genet..

[6] Aljazi M.B., Gao Y., Wu Y., He J. (2022). SMYD5 is a histone H3-specific methyltransferase mediating mono-methylation of histone H3 lysine 36 and 37. Biochem. Biophys. Res. Commun..

[7] Zhang Y., Fang Y., Tang Y., Han S., Jia J., Wan X., Chen J., Yuan Y., Zhao B., Fang D. (2022). SMYD5 catalyzes histone H3 lysine 36 trimethylation at promoters. Nat. Commun..

[8] Stender J.D., Pascual G., Liu W., Kaikkonen M.U., Do K., Spann N.J., Boutros M., Perrimon N., Rosenfeld M.G., Glass C.K. (2012). Control of proinflammatory gene programs by regulated trimethylation and demethylation of histone H4K20. Mol. Cell.

[9] Boehm D., Lam V., Schnolzer M., Ott M. (2023). The lysine methyltransferase SMYD5 amplifies HIV-1 transcription and is post-transcriptionally upregulated by Tat and USP11. Cell Rep..

[10] Park J., Wu J., Szkop K.J., Jeong J., Jovanovic P., Husmann D., Flores N.M., Francis J.W., Chen Y.C., Benitez A.M. (2024). SMYD5 methylation of rpL40 links ribosomal output to gastric cancer. Nature.

[11] Miao B., Ge L., He C., Wang X., Wu J., Li X., Chen K., Wan J., Xing S., Ren L. (2024). SMYD5 is a ribosomal methyltransferase that catalyzes RPL40 lysine methylation to enhance translation output and promote hepatocellular carcinoma. Cell Res..

[12] Hamey J.J., Shah M., Wade J.D., Bartolec T.K., Wettenhall R.E.H., Quinlan K.G.R., Williamson N.A., Wilkins M.R. (2025). SMYD5 is a ribosomal methyltransferase that trimethylates RPL40 lysine 22 through recognition of a KXY motif. Cell Rep..

[13] Shago M., Giguere V. (1996). Isolation of a novel retinoic acid-responsive gene by selection of genomic fragments derived from CpG-island-enriched DNA. Mol. Cell. Biol..

[14] Calpena E., Palau F., Espinos C., Galindo M.I. (2015). Evolutionary History of the Smyd Gene Family in Metazoans: A Framework to Identify the Orthologs of Human Smyd Genes in Drosophila and Other Animal Species. PLoS ONE.

[15] Schubert H.L., Blumenthal R.M., Cheng X. (2003). Many paths to methyltransfer: A chronicle of convergence. Trends Biochem. Sci..

[16] Ensemble of Gene Set Enrichment Analyses Online. https://useast.ensembl.org/Homo_sapiens/Gene/Summary?db=core;g=ENSG00000135632;r=2:73214222-73227221.

[17] Kidder B.L., Hu G., Cui K., Zhao K. (2017). SMYD5 regulates H4K20me3-marked heterochromatin to safeguard ES cell self-renewal and prevent spurious differentiation. Epigenet. Chromatin.

[18] Zhang Y., Alshammari E., Sobota J., Yang A., Li C., Yang Z. (2022). Unique SMYD5 Structure Revealed by AlphaFold Correlates with Its Functional Divergence. Biomolecules.

[19] Xu S., Wu J., Sun B., Zhong C., Ding J. (2011). Structural and biochemical studies of human lysine methyltransferase Smyd3 reveal the important functional roles of its post-SET and TPR domains and the regulation of its activity by DNA binding. Nucleic Acids Res..

[20] Abu-Farha M., Lanouette S., Elisma F., Tremblay V., Butson J., Figeys D., Couture J.F. (2011). Proteomic analyses of the SMYD family interactomes identify HSP90 as a novel target for SMYD2. J. Mol. Cell Biol..

[21] Choudhury N.R., Heikel G., Trubitsyna M., Kubik P., Nowak J.S., Webb S., Granneman S., Spanos C., Rappsilber J., Castello A. (2017). RNA-binding activity of TRIM25 is mediated by its PRY/SPRY domain and is required for ubiquitination. BMC Biol..

[22] Castello A., Fischer B., Eichelbaum K., Horos R., Beckmann B.M., Strein C., Davey N.E., Humphreys D.T., Preiss T., Steinmetz L.M. (2012). Insights into RNA biology from an atlas of mammalian mRNA-binding proteins. Cell.

[23] Vakoc C.R., Sachdeva M.M., Wang H., Blobel G.A. (2006). Profile of histone lysine methylation across transcribed mammalian chromatin. Mol. Cell. Biol..

[24] Liang X., Zuo M.Q., Zhang Y., Li N., Ma C., Dong M.Q., Gao N. (2020). Structural snapshots of human pre-60S ribosomal particles before and after nuclear export. Nat. Commun..

[25] Williamson N.A., Raliegh J., Morrice N.A., Wettenhall R.E. (1997). Post-translational processing of rat ribosomal proteins. Ubiquitous methylation of Lys22 within the zinc-finger motif of RL40 (carboxy-terminal extension protein 52) and tissue-specific methylation of Lys4 in RL29. Eur. J. Biochem..

[26] Holm M., Natchiar S.K., Rundlet E.J., Myasnikov A.G., Watson Z.L., Altman R.B., Wang H.Y., Taunton J., Blanchard S.C. (2023). mRNA decoding in human is kinetically and structurally distinct from bacteria. Nature.

[27] Isel C., Karn J. (1999). Direct evidence that HIV-1 Tat stimulates RNA polymerase II carboxyl-terminal domain hyperphosphorylation during transcriptional elongation. J. Mol. Biol..

[28] Fujii T., Tsunesumi S., Sagara H., Munakata M., Hisaki Y., Sekiya T., Furukawa Y., Sakamoto K., Watanabe S. (2016). Smyd5 plays pivotal roles in both primitive and definitive hematopoiesis during zebrafish embryogenesis. Sci. Rep..

[29] Kidder B.L., He R., Wangsa D., Padilla-Nash H.M., Bernardo M.M., Sheng S., Ried T., Zhao K. (2017). SMYD5 controls heterochromatin and chromosome integrity during embryonic stem cell differentiation. Cancer Res..

[30] Jin X., Liu W., Miao J., Tai Z., Li L., Guan P., Liu J.X. (2021). Copper ions impair zebrafish skeletal myofibrillogenesis via epigenetic regulation. FASEB J..

[31] Fellous A., Earley R.L., Silvestre F. (2019). The Kdm/Kmt gene families in the self-fertilizing mangrove rivulus fish, Kryptolebias marmoratus, suggest involvement of histone methylation machinery in development and reproduction. Gene.

[32] Song J., Liu Y., Chen Q., Yang J., Jiang Z., Zhang H., Liu Z., Jin B. (2019). Expression patterns and the prognostic value of the SMYD family members in human breast carcinoma using integrative bioinformatics analysis. Oncol. Lett..

[33] Meng X., Zhao Y., Liu J., Wang L., Dong Z., Zhang T., Gu X., Zheng Z. (2019). Comprehensive analysis of histone modification-associated genes on differential gene expression and prognosis in gastric cancer. Exp. Ther. Med..

[34] Chi G., Pei J., Li X., Li X., Pang H., Cui J., Wu D., Qu G., He Y. (2022). SMYD5 acts as a potential biomarker for hepatocellular carcinoma. Exp. Cell Res..

[35] Hu M., Chen S., Zhen Y., Wang X., Zhong Y., Liang X., Chong C.M., Zhong H.J. (2025). SMYD5-BRD4 Interaction Drives Hepatocellular Carcinoma Progression: A Combined in Silico and Experimental Analysis. Pharmaceuticals.

[36] Conrad R.J., Fozouni P., Thomas S., Sy H., Zhang Q., Zhou M.M., Ott M. (2017). The Short Isoform of BRD4 Promotes HIV-1 Latency by Engaging Repressive SWI/SNF Chromatin-Remodeling Complexes. Mol. Cell.

[37] Tae I.H., Ryu T.Y., Kang Y., Lee J., Kim K., Lee J.M., Kim H.W., Ko J.H., Kim D.S., Son M.Y. (2024). Negative regulation of SH2B3 by SMYD5 controls epithelial-mesenchymal transition in lung cancer. Mol. Cells.

[38] Erdem J.S., Zavodna T., Ervik T.K., Skare O., Hron T., Anmarkrud K.H., Kusnierczyk A., Catalan J., Ellingsen D.G., Topinka J. (2023). High aspect ratio nanomaterial-induced macrophage polarization is mediated by changes in miRNA levels. Front. Immunol..

[39] Zhang Y., Hayden S., Spellmon N., Xue W., Martin K., Muzzarelli K., Kovari L., Yang Z. (2021). Sperm chromatin-condensing protamine enhances SMYD5 thermal stability. Biochem. Biophys. Res. Commun..

[40] Rathke C., Baarends W.M., Awe S., Renkawitz-Pohl R. (2014). Chromatin dynamics during spermiogenesis. Biochim. Biophys. Acta.

[41] Hammoud S.S., Nix D.A., Zhang H., Purwar J., Carrell D.T., Cairns B.R. (2009). Distinctive chromatin in human sperm packages genes for embryo development. Nature.

[42] Rafnsdottir S., Jang K., Halldorsdottir S.T., Vinod M., Tomasdottir A., Moller K., Halldorsdottir K., Reynisdottir T., Atladottir L.H., Allison K.E. (2024). SMYD5 is a regulator of the mild hypothermia response. Cell Rep..

[43] Hou Y., Sun X., Gheinani P.T., Guan X., Sharma S., Zhou Y., Jin C., Yang Z., Naren A.P., Yin J. (2022). Epithelial SMYD5 Exaggerates IBD by Down-regulating Mitochondrial Functions via Post-Translational Control of PGC-1alpha Stability. Cell Mol. Gastroenterol. Hepatol..

[44] Abraham C., Cho J.H. (2009). Inflammatory bowel disease. N. Engl. J. Med..

[45] Xiao C., Su Z., Zhao J., Tan S., He M., Li Y., Liu J., Xu J., Hu Y., Li Z. (2025). Novel regulation mechanism of histone methyltransferase SMYD5 in rheumatoid arthritis. Cell Mol. Biol. Lett..

[46] Smolen J.S., Aletaha D., McInnes I.B. (2016). Rheumatoid arthritis. Lancet.

[47] Nemeth T., Nagy G., Pap T. (2022). Synovial fibroblasts as potential drug targets in rheumatoid arthritis, where do we stand and where shall we go?. Ann. Rheum. Dis..

[48] Bartok B., Firestein G.S. (2010). Fibroblast-like synoviocytes: Key effector cells in rheumatoid arthritis. Immunol. Rev..

[49] Hickenlooper S.M., Davis K., Szulik M.W., Sheikh H., Miller M., Valdez S., Bia R., Franklin S. (2022). Histone H4K20 Trimethylation Is Decreased in Murine Models of Heart Disease. ACS Omega.

[50] Hickenlooper S., Brady C., Bia R., Visker J.R., Wang L., Valdez S., Gwynn C., Roland M.N., Kyriakopoulos C.P., Sideris K. (2025). Expression profiles of histone H4K20 methylation and its associated enzymes in mouse cardiac disease and human heart failure. Epigenetics.

[51] Baker B.H., Freije S., MacDonald J.W., Bammler T.K., Benson C., Carroll K.N., Enquobahrie D.A., Karr C.J., LeWinn K.Z., Zhao Q. (2024). Placental transcriptomic signatures of prenatal and preconceptional maternal stress. Mol. Psychiatry.

[52] Tracy C., Warren J.S., Szulik M., Wang L., Garcia J., Makaju A., Russell K., Miller M., Franklin S. (2018). The Smyd Family of Methyltransferases: Role in Cardiac and Skeletal Muscle Physiology and Pathology. Curr. Opin. Physiol..

[53] Wang Z., Schwartz R.J., Liu J., Sun F., Li Q., Ma Y. (2021). Smyd1 Orchestrates Early Heart Development Through Positive and Negative Gene Regulation. Front. Cell Dev. Biol..

[54] Warren J.S., Tracy C.M., Miller M.R., Makaju A., Szulik M.W., Oka S.I., Yuzyuk T.N., Cox J.E., Kumar A., Lozier B.K. (2018). Histone methyltransferase Smyd1 regulates mitochondrial energetics in the heart. Proc. Natl. Acad. Sci. USA.

[55] Anderson A., Miller M., Wang L., Franklin S. (2016). Smyd5 Regulates Hypertropic Growth in the Heart. FASEB J. Physiol..

